# Nonsurgical Medical Aesthetics and Patient Quality of Life: An Umbrella Review

**DOI:** 10.1093/asjof/ojae096

**Published:** 2024-10-30

**Authors:** Barbara Hemsworth, Cody Hemsworth, Sarah A Richmond

## Abstract

Nonsurgical cosmetic facial procedures have become popular treatment options for individuals seeking aesthetic improvements. Despite a breadth of literature on patient satisfaction with treatment outcomes, there is a lack of information specific to changes in quality of life outcomes. The objective of this umbrella review is to report the effectiveness of nonsurgical facial aesthetic treatments on reported quality of life in cosmetic treatment–seeking patients. The authors also aim to identify gaps in the literature on measures of quality of life outside of patient satisfaction. The authors completed a comprehensive, systematic search of review articles across 6 databases, including Medline, CINAHL Plus, EMBASE, APA PsycINFO, Cochrane Reviews, and Google Scholar. They included review-level studies that examine the changes in quality of life measures following treatment. A critical appraisal was completed for each review article included. A total of 7 reviews were included. One review was of strong quality, 2 moderate, and 4 were weak. Several nonsurgical procedures were evaluated across reviews, including injectable neurotoxins, dermal fillers, and laser skin resurfacing. The majority of included studies reported increases in measures of quality of life, posttreatment in the same patient or compared with controls. The most commonly reported measure was psychological well-being, followed by self-perception. There was a lack of measures outside of improvements to aesthetics, including those specific to mental health (eg, depression). Overall, based on the studies on nonsurgical treatments, the authors report increases in overall quality of life. This conclusion should be interpreted with caution, as the majority of reviews included were of moderate-to-weak quality. A major gap in this literature includes mental health outcomes. Future research should focus on increasing the rigor of reporting for systematic reviews.

**Level of Evidence: 2 (Risk):**

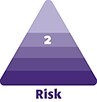

Nonsurgical cosmetic facial procedures have become popular treatment options for individuals seeking aesthetic improvements. In the United States, it is estimated that these treatments generate ∼16.5 billion dollars annually, with a 228% increase in the number of nonsurgical facial procedures reported from 2000 to 2018, according to The Aesthetic Society.^1^ In Canada, there is a lack of data specific to the number of patients seeking noninvasive facial treatments; however, it is estimated that over half a million cosmetic enhancement procedures were conducted in 2005 across the country, according to the Cosmetic Surgery Statistics Canada Report. In this report, a 19% increase in nonsurgical rhytidectomy procedures was observed in 2005, concomitantly with the number of surgical treatments decreasing. Anecdotally, there have been increased reports of requests for nonsurgical procedures since the start of the COVID-19 pandemic in Canada. This is hypothesized to be due to increased participation in virtual meetings and reflection of one's self-image.

Nonsurgical cosmetic treatments are often grouped into treatment categories that include injectable neurotoxins, injectable dermal fillers, and laser skin resurfacing. The most popular of which includes injection of Botulinum neurotoxin type A, used worldwide.^2^ Patients seeking cosmetic treatments range in their desired outcomes, including improvements in their physical appearance and/or specific treatments for existing inflammatory disorders of the skin, including acne vulgaris and rosacea.

There are several review-level publications on changes in patient satisfaction with cosmetic treatments. For example, in a study by Radelusco et al, of the 674 patients included across 8 primary studies on patient satisfaction after nonsurgical rhinoplasty using hyaluronic acid, over 96% of patients reported being satisfied or very satisfied with their treatment.^3^ Further, in the review by Fagien and Carruthers, patient satisfaction post-Botulinum toxin type A treatment was reported consistently and significantly high. Depending on treatment area and dose, patient satisfaction ranged from 65% to >90%.^4^ Despite the increase in the number of Canadians seeking treatment and data specific to patient satisfaction, there is a lack of data specific to patient's mental and emotional health, social, and occupational well-being.^5^ Well-being is a complex and multifaceted construct that includes psychological and social dimensions that individuals characterize of themselves and their lives. Quality of life measures capture well-being across several dimensions, including mental and physical health, such as anxiety, stress, and disability status.^6^ Further, it has been speculated that patient satisfaction and patient quality of life are not correlated and cannot be used to estimate the effectiveness of other measures including psychological and psychosocial well-being.^5,7^ There is increasing evidence to support that psychological well-being is associated with lower disease risk; thus, understanding more on these measures and their contribution to health risk is prudent.^6^ Further, reporting on these specific outcomes can be used to suit patient treatment choice, screen and stratify patients, and pretreatment.^8^ Finally, a more fulsome understanding of the changes to patient quality of life with cosmetic treatments can serve as foundational metrics in future studies.

The primary objective of this umbrella review was to report the effectiveness of nonsurgical facial aesthetic treatments on quality of life in cosmetic treatment seeking patients. Secondary objectives were to identify gaps in the existing literature on specific measures of quality of life outside of patient satisfaction.

## METHODS

This umbrella review was completed using guidance from Pollock et al.^9^ Our research question focused on any population seeking nonsurgical aesthetic treatments on any change in quality of life measure. Our methodology also followed the Preferred Reporting Items for Systematic Reviews and Meta-Analyses (PRISMA) guideline for reporting systematic reviews.^10^ Ethical approval was not required for this study as the data were derived from previously published studies.

### Search Strategy and Inclusion/Exclusion Criteria

We used several search terms and combinations of search terms across 6 databases to identify relevant articles (Table 1). Two independent reviewers screened article titles and abstracts for inclusion. We also applied hand search methodology to the included studies as well as consultation with experts in the field (B.H., C.H.) to ensure data capture. Medline, CINAHL Plus, EMBASE, APA PsycINFO, Cochrane Reviews, and Google Scholar were searched from October 1, 2018 to October 1, 2023. The following inclusion criteria were applied to each database search: examined the relationship between a nonsurgical aesthetic treatment and quality of life measures on any population; articles published in English; and any literature review study design (systematic review, literature review, scoping review, narrative reviews, etc). Articles were excluded if they were studies that: measured only patient satisfaction with aesthetic treatments; examined only surgical interventions; measured only pain or pain-related outcomes; compared 1 treatment to another aesthetic treatment; measured nonfacial aesthetic treatments; measured outcomes in special populations (eg, cancer and HIV). Nonreview study designs were also excluded (eg, primary studies and review study protocols). All duplicate titles were removed before title and abstract screening.

**Table 1.** MeSH Headings and Key Word Search Terms, by Population, Exposure, and Outcome

Population	Exposures	Outcome
No limits	Medical aesthetics	Quality of life
	Aesthetics	Self-esteem
	Facial aesthetics	Self-confidence
	Botox (Allergan, Dublin, Ireland)	Patient outcomes
	Botulinum neurotoxin type A	Psychological well-being
	Onabotulinumtoxin A	Social well-being
	Abobotulinumtoxin A	Physical well-being
	Incobotulinumtoxin A	Occupational well-being
	Daxibotulinumtoxin A	Mental health
	Prabotulinumtoxin A	
	Dermal filler	
	Hyaluronic acid	
	Cosmetic laser treatments	
	Laser treatments	

### Data Extraction and Critical Appraisal

Relevant data from each review included in this study were extracted by 1 reviewer. A second reviewer performed a 20% data extraction review to ensure accuracy. We performed a double, independent critical appraisal (CA) of each included article using the Health Evidence Quality Appraisal Checklist, used specifically for systematic reviews.^11^ The Health Evidence Quality Appraisal Checklist is designed to appraise systematic reviews and meta-analyses to determine the effectiveness of interventions. Questions are assigned a point value (1,0) based on a yes/no answer. Responses are summed across the checklist to determine a final score for each study, indicative of the quality rating of the review. Questions are focused around key elements of CA, including research question, inclusion/exclusion criteria, search strategy and dates, level of evidence, methodological quality, results, analysis and appropriateness of analytic methods, and interpretation.^11^ The final score out of a possible 10 is then assigned a rating: strong (8-10), moderate (5-7), and weak (0-4).^11^ If there were discrepancies in the CA process, they were discussed and resolved by a third, independent reviewer.

### Data Synthesis and Analyses

We present a narrative synthesized data analysis, grouping review studies by treatment type. For studies that included >1 treatment, we separated each study into appropriate treatment groupings. Treatments were grouped into the following categories: Botulinum toxin type A (including Onabotulinumtoxin A, Abobotulinumtoxin A, Incobotulinumtoxin A, Daxibotulinumtoxin A, and Prabotulinumtoxin A); dermal fillers (including absorbable, nonabsorbable, and augmentation fillers); and laser skin resurfacing (including laser peels, laser vaporization, and lasabrasion). Due to the variability in outcome measures used and the heterogeneity of review study methodology, a meta-analysis was not possible.

## RESULTS

**Figure 1.**
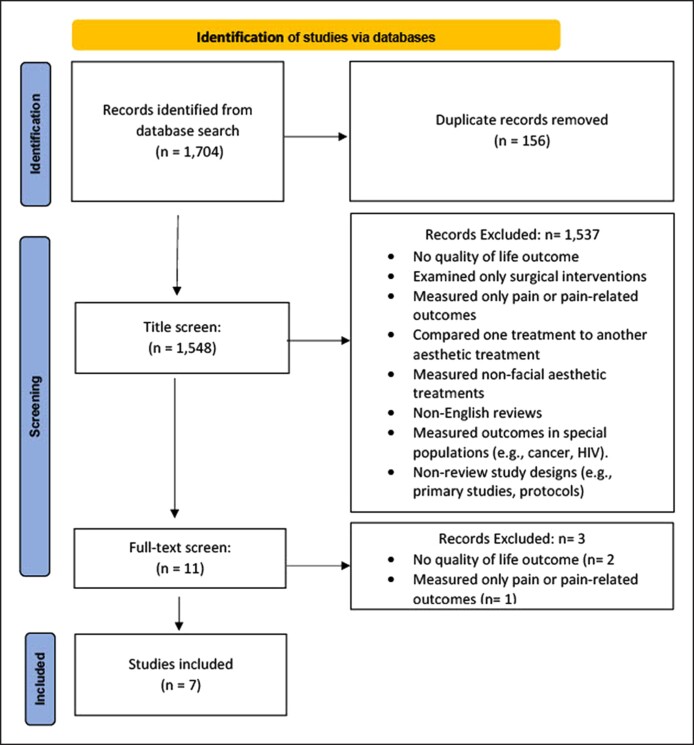
Preferred Reporting Items for Systematic Reviews and Meta-Analyses diagram.

A total of 1548 review articles were screened from the 6 databases used for title search. A total of 11 studies were reviewed in full-text form, leaving a remaining 7 review studies that met inclusion criteria (Supplemental Table 1). Four studies were excluded at the full-text review stage, due to a lack of specific quality of life outcomes. A detailed description of the review process is shown in Figure 1, using guidance from PRISMA.^10^ A total of 6 studies were systematic reviews, and 1 was a literature review. All studies included nonsurgical medical aesthetic treatments,^5,12–15^ 2 studies included both surgical and nonsurgical treatment measures.^8,16^ Study characteristics including patient populations, treatment (intervention), comparison groups, outcomes, study findings, and limitations are found in Supplemental Table 1.

There were a number of tools used to assess quality of life in patients across primary studies in each systematic review. The majority of primary studies included across reviews used the FACE-Q outcome appraisal tool, second to versions of the World Health Organization—Quality of Life (WHO-QOL) tool. Other tools used included the Global Aesthetic Improvement Scale (GAIS), Facial Lines Treatment Satisfaction (FTS), Facial Line Outcomes (FLO), Service Provision Assessment (SPA), Health Measurement (HM) Questionnaires, and the Quality of Life Enjoyment and Satisfaction Questionnaire. In 1 review, the authors reported studies that self-developed an observer-reported outcome evaluation tool.^16^

The FACE-Q, the tool most reported across studies, is a widely used tool in cosmetic treatment studies that assess the change in quality of life outcomes and treatment-specific decisional satisfaction in patients.^17^ There are 5 scales included in the tool: psychological well-being, social function (that includes social confidence), treatment decision satisfaction, treatment outcome satisfaction, early life impact of treatment, and a specific checklist for treatment recovery.^17^

There were a total of 225 primary studies included across reviews. Of those, 102 studies included specific quality of life measures. For those studies that reported on Botulinum toxin type A, studies consistently reported positive changes across quality of life measures. This included psychological, self-perception, relationships, and age-appraisal measures. Studies that reported on dermal fillers demonstrated mixed results with 1 review reporting varying levels of effectiveness and the other reporting positive improvements without reporting specific data from the primary studies. Studies that reported on laser skin resurfacing report increased measures of quality of life; however, this review did not present individual level data nor specifically link quality of life measures with laser resurfacing specifically. Finally, reviews that examined combination treatments and other treatment modalities reported null results.

### Study Quality

Overall, studies included in this review were of weak reporting quality. One review was rated as strong, 2 were of moderate quality, and 4 studies were of weak-reporting quality. Table 2 reports the specific results of the CA.

**Table 2.** Critical Appraisal of Included Reviews (*n* = 7) Using the Health Evidence Tool for Systematic Reviews

Health Evidence Checklist Item	Cohen and Scuderi (2017)	Galadari et al (2021)	Hoffman and Fabi (2022)	Imadojemu et al (2013)	Ou et al (2023)	Shah and Rieder (2021)	Wang and Rieder (2019)
1. Is the research question clearly focused, describing the population, intervention, comparison and outcome(s) of interest?	1	1	1	1	1	1	1
2. Are appropriate criteria used to select studies to include in the review?	0	1	0	1	1	0	1
3. Is the search strategy comprehensive and reproducible?	0	1	0	1	1	0	1
4. Does the search strategy cover an adequate number of years?	1	1	0	1	1	0	1
5. Is the level of evidence of studies included in the review described?	0	0	0	1	0	0	0
6. Are included studies rigorously assessed for risk of bias/methodological quality and reported on?	0	0	0	1	1	0	1
7. Are the quality assessments completed in duplicate with a method for conflict resolution described?	0	0	0	1	0	0	0
8. Are the methods used to compare and/or combine results across studies appropriate?	0	0	0	1	1	0	1
9. Are study quality and level of evidence taken into consideration when interpreting results?	0	0	0	0	0	0	0
10. Is the certainty of the review's conclusions supported by the methodological approach and review findings?	0	0	0	1	0	0	1
Total score	2	4	1	9	6	1	7

Scores: ≥8 = strong; 5-7 = moderate; ≤ 4 = weak.^11^

### Botulinum Toxin Type A and Abobotulinumtoxin A Treatments

#### Psychological Improvement

In a report by Cohen and Scuderi, 5 studies examined the effect of Botulinum toxin type A and Abobotulinumtoxin A on quality of life outcomes. In 2 studies within this review, the authors reported psychological improvements in patients posttreatment. In 1 study, they found that those with BoNT-A had a significantly more positive mood than those without, through lower anxiety and depression scores, and 1 study found greater psychological improvement and age-related impact, improved perception of crow's feet line appearance, and treatment satisfaction compared with placebo.^12^ In a report by Hoffman and Fabi, 2 of 3 studies measuring changes in psychological functioning reported statistically significant increases compared with pretreatment using the FACE-Q quality of life questions.^14^ Imadojemu et al reported only 1 study with noninvasive treatment (BoNT-A) and reported increases in self-esteem compared with placebo group.^8^ Finally, Wang and Rieder reported overall positive effects on affect, mood, and self-confidence.^5^

#### Self-Perception

Two reviews reported on changes in self-perception.^5,14^ In Hoffman and Fabi, the majority of studies report treatment significantly improved patient self-perception in studies using both pre- and postdesigns as well as treatment vs placebo. For example, in 1 study, the authors demonstrated treatment of glabellar lines with Botulinum toxin type A resulted in a high level of patient satisfaction and corresponded to a more positive self-perception 4 months after injection.^14^ Further, in another study, the authors demonstrated that a full-face approach using variable doses led to significant improvements in reported self-assessment, self-image, and level of satisfaction compared with baseline.^14^ In Wang and Rieder, all studies included reported increases in patient-reported appearance, youthfulness, and perception of attractiveness.

#### Physical, Psychological, and Social Relationships

Galadari et al reported on 1 primary study that examined changes in physical, psychological, and social relationship scores between baseline and 4 week posttreatment scores in patients treated with Abobotulinumtoxin A. In this study, the authors reported significant improvements in the medium dose groups (166-205 units) compared with both low (120-165 units) and high (206-250 units) unit dose groups.^13^ These measures were reported using the World Health Organization Quality of Life—Brief Version.

#### Age Appraisal and Satisfaction

In 3 studies, Hoffman and Fabi reported on changes in patient's appraisal of their age and satisfaction. In 2 of the 3 reported studies, the authors indicated a decrease in age-perception-related measures.^14^

### Dermal Filler Treatments

Hoffman and Fabi reported on changes to measures of quality of life using the FACE-Q tool in dermal filler patients, compared with scores pretreatment. In 2 studies in this review, the authors reported on psychological well-being and reported mixed results. In 1 study, they reported no change in scores posttreatment and in the other, they reported nonstatistically significant increases at 3 and 12 months.^14^ Ou et al reported on 8 studies that examined the change in quality of life pre- to posttreatment on nonsurgical chin augmentation using hyaluronic acid dermal filler. All studies reported increases in both physical and psychological well-being modules, as measured by the FACE-Q; however, this review did not specifically report individual study results for quality of life measures.^15^

### Laser Resurfacing Treatments

One review included results specific to laser resurfacing treatment. Imadojemu et al reported significant improvement from baseline to 6 months posttreatment, using the HM Questionnaire; however, review authors did not present individual level data nor specifically comment on laser resurfacing as a treatment modality.

### Combination and Other Treatments

Two reviews included primary studies that examined combination treatments.^5,14^ Both studies reported that multiple facial areas or with multiple treatment modalities may improve the quality of outcomes further than isolated treatments. For example, in a study by Hoffman and Fabi, 2 of the 4 studies measuring psychological well-being reported statistically significant increases posttreatment and 2 of the 3 studies measuring social functioning and social confidence reported statistically significant results from baseline.

In 2 studies, Hoffman and Fabi examined changes in age appraisal with platelet-rich plasma treatment and threading absorbable suspension suture treatment, compared with pretreatment. Both studies reported nonsignificant decreases in age appraisal (decreased perception of individual age) from participating patients.^14^

One study included in this review reported on both invasive and noninvasive treatments, reporting qualitative results.^16^ Noninvasive treatments included botulinum toxin, calcium hydroxyapatite, hyaluronic acid, deoxycholic acid, and fat injection as well as laser skin resurfacing. Study authors analyzed data from each study using thematic analyses and reported on changes to quality of life measures, including perception changes in age, attractiveness, sociability, relationship success, and occupational and financial competency.^16^ This study, however, was reported weak from the CA process, as this study lacked specificity between treatment and outcomes, did not include any CA of included studies, and reported overly simplified results that did not allow for appropriate synthesis.

## DISCUSSION

Of the 102 primary studies included across 7 systematic reviews, the majority of studies reported increases to quality of life measures in patients seeking nonsurgical cosmetic treatments. The overall quality of reporting for these reviews, however, was weak. All reviews but 2 focused on nonsurgical procedures. The studies that included specific surgical techniques were outside the scope of this umbrella review.^8,16^ The majority of reviews examined studies on the effectiveness of Botox (Allergan, Dublin, Ireland) (or affiliate) on the change in quality of life measures. For example, of the 7 included reviews, only 1 review did not report on Botox as a treatment option.^15^ The specific research question in this review was to examine patient satisfaction with nonsurgical chin augmentation using hyaluronic acid.

We aimed to examine the existing scientific evidence on the effectiveness of cosmetic treatments on measures of quality of life. This was to address the current gap in the literature of these specific measures, over the breadth of literature on patient satisfaction. As stated by Jandhyala, there is a lack of data on the correlation between patient satisfaction and health-related quality of life measures.^7^ These measures are the most used and referenced patient-reported outcomes that describe and define states of individual health, focused on overall well-being that includes physical, mental, and social components. The benefit to using health-related quality of life measures, over measures such as patient satisfaction, is the ability to measure patient responsivity to specific interventions over time, as well as facilitate shared treatment decision making between patient and practitioner.^18^ This provides the opportunity to tailor specific treatments based on specific health-related quality of life outcomes, focusing on patient needs.

Overall, there was significant variability in the tools used to assess changes in quality of life outcomes across reviews including the FACE-Q, as well as several Likert-type scales used to determine levels of satisfaction, posttreatment. Further, the majority of studies did not evaluate the validity of the tools used. The FACE-Q outcome appraisal tool was the most cited tool in the included primary studies. This was followed by several other tools, including the WHO-QOL tool, the GAIS, FTS, FLO, SPA, and Quality of Life Enjoyment and Satisfaction Questionnaire, among others. Future research should use a validated, reliable tool to assess quality of life measures that can also be used to pool effect estimates across studies. This would allow for a meta or network analysis to be completed to compare the clinical effectiveness of treatments across studies, to increase sample size, and power to detect meaningful differences. This would provide practitioners with both quantitative and qualitative measures to inform treatment choices.

Significant data gaps were noted in the studies evaluated in this review. This included a lack of mental health outcomes such as depression or anxiety, occupational specific quality of life outcomes, data specific to men, and data reporting measures across differing races and/or ethnicities. It is important to include measures of mental health when examining changes posttreatment as to quantify increases or decreases. There is literature in this area specific to invasive procedures which reports increases in patient perceptions of body image but mixed results on measures of self-esteem, anxiety, and depression postsurgical intervention.^19^ The evaluation of these measures in the noninvasive procedure space is necessary. Further, there is a lack of published studies that examine the effectiveness of combination treatments, including multiple facial areas or with multiple treatment modalities on quality of life outcomes. Finally, studies considering the effect of other important baseline measures in the reported change to quality of life measures are required to decrease measurement bias. For example, consideration of a patient’s educational attainment, sex, and age among other important variables plays a significant role in determining the direction and magnitude of quality of life changes, posttreatment.

Our review reports consistent findings across included studies, despite significant concerns in methodological quality, reporting, and reproducibility. We believe, however, an increase in the rigor of primary studies examining health-related quality of life outcomes in cosmetic treatment patients would provide a new narrative on the benefit of nonsurgical cosmetic treatments on both mental and physical health.

### Strengths and Limitations

We note several strengths to this review. First, we completed a comprehensive, reproducible search strategy across 6 relevant databases, including a gray literature search. Second, we included all review types, all nonsurgical treatment types. Finally, we completed a rigorous CA process using a tool specific to systematic reviews.

This review, however, has several limitations; most of which are due to the reporting of review papers themselves. First, none of the included reviews were categorized as strong quality. The majority of reviews lacked commentary on internal validity, specifically the rigor of analyses performed in each primary study. Without confidence in the study design, data collection, and analytic approach, as well as interpretation of effect estimates presented, it is difficult to effectively summarize results across studies. Further to this point, several reviews did not complete a CA process in their syntheses; therefore, the risk of bias can be high for studies included. Interpreting results without the support of levels of evidence can produce erroneous conclusions. Second, the majority of reviews did not comment specifically on individual sample and control groups (where relevant), on the reliability and validity of the tool used to measure quality of life, and on generalizability to other study populations. Third, 2 reviews did not provide a descriptive summary of the patient populations included.^5,14^ This information is a necessary component of the review process to comment on the external validity of study results. Further, 5 reviews did not report on the jurisdictions included in the primary studies.^5,12,14–16^ Of the reviews that did, the majority were conducted in the United States and Canada, thus limiting the generalizability of results to other populations. Fourth, several studies did not include description of baseline measures, only changes in measures, posttreatment, or compared with controls. Finally, there is known measurement bias in studies that utilize self-report. Survey data are quite ubiquitous for social desirability bias, particularly accompanied by sample bias. In this specific patient population (eg, cosmetic treatment patients), there may be systematic misreporting given the impetus of these patients seeking nonsurgical treatment options.

## CONCLUSIONS

This umbrella review aimed to synthesize and critically appraise the existing evidence on the effectiveness of noninvasive aesthetic treatments on quality of life outcomes. Overall, there is a lack of quality, synthesized data on these specific treatments with quality of life outcomes outside of patient satisfaction. More research is needed to better understand the association between treatments and these outcomes. Of the 7 reviews included, the majority of studies reported increases to measures of quality of life including psychological well-being and self-perception; however, there was a lack of measures specific to quality of life outside of improvements to aesthetics, including changes specific to mental health (eg, anxiety and depression). This review provides synthesized, critically appraised information from the existing scientific literature specific to quality of life outcomes and outlines current gaps in our knowledge. Future work in this area should focus on an increase in the rigor of reporting and measure changes to other health outcomes, including mental and physical health outcomes that can examine the effectiveness of treatment options.

## Supplemental Material

This article contains supplemental material located online at https://doi.org/10.1093/asjof/ojae096.

## Supplementary Material

ojae096_Supplementary_Data
