# Supplementary material for: Nonsurgical Medical Aesthetics and Patient Quality of Life: An Umbrella Review
Source: Aesthet Surg J Open Forum. 2024 Oct 30;6:ojae096. doi: 10.1093/asjof/ojae096 (PMC11646117; doi:10.1093/asjof/ojae096)
Supplement: ojae096_Supplementary_Data [file ojae096_supplementary_data.docx]

| **Supplementary Table 1.** Characteristics of included reviews (n=7) | | | | | | | |
| --- | --- | --- | --- | --- | --- | --- | --- |
| Source (Year) | Study Type | Objective | Included Studies, Jurisdiction | Population | Treatment(s) | Comparison, Quality of Life Measure (QoL) | Findings, Limitations |
| Cohen & Scuderi (2017) | Systematic Review | To review the safety of BoNT-A in the treatment of glabellar lines, other areas of the face, and scar optimization, and to review data supporting patient satisfaction | Total (N=86 studies); 5 measured quality of life (QoL)  Jurisdiction:  Not reported | N=557 (527 women, 31 men)  Age=~mean 53 years, 1 study range; 13-49 years | AbobotulinumtoxinA (ABO) (several sites across studies) | Pre-treatment and controls  QoL Measure:  Did not report specifc measures used in included studies | ABO treatment consistently and significantly associated with high patient QoL including self-perception, relative age, positive mood (via lower anxiety and depression), psychological improvement, and self-assessment  Limitations:  Only reviewed two data bases; No critical appraisal (CA) process; No double title screen or data extraction (DE); Did not report individual study results |
| Galadari et al. (2021) | Systematic Review | To assess the  evidence regarding treatment approaches, efficacy, safety and patient-reported outcomes relating to aboBoNT-A for cosmetic treatment for treatment of the neck and of middle and lower areas of the face | Total (N=10 studies)  Jurisdiction:  N=3 (Brazil), N=2 (Lebanon), N=2 (United States- US), N=1 (each South  Korea, Taiwan, Thailand) | N= 10 - 383, Age=32.8 - 55.9 years, Female= 93% -100% (of reported) | Botox injections (N=6) middle and lower face, N=2 masseter, N=1  gingival display (gummy smile), N=1 platysma muscle only | Pre-post treatment  QoL Measures: World Health Organization Quality of Life—Brief Version questionnaire (WHOQOL-BREF) | There was a significant increase in physical QoL between baseline and 4 weeks. When comparing total dose groups, a medium dose group (166–205 U) had significantly better physical, psychological and social relationship QoL scores than the low (120–165 U) and high (206–250 U) dose groups  Limitations:  No CA process; No double title screen or DE; Small number of included studies |
| Hoffman & Fabi (2022) | Literature Review | To review clinical trials of minimally invasive (non-surgical) aesthetic  facial procedures to determine the impact of treatment on satisfaction with appearance,  psychological wellbeing, age appraisal, and satisfaction | Total (N=31 studies); 12 measured QoL  Jurisdiction:  Not reported | Not reported | Facial filler (upper, mid, and lower face), lip filler, botox, combined treatment (facial  filler and botox), and other | Pre-post treatment  QoL Measure:  FACE-Q | Mid-face & nasolabial fold dermal filler: No QoL  Chin/lower face filler: N=2 psychological wellbeing: 1 study: 15.4 change from baseline at 3 months, ~15.0 at 12 months, 2nd study: 13.4 at 3 months, 9.1 at 12 months (all non-significant)  Lip filler studies: No QoL; Botox studies: N=4 psychological functioning (3 studies): reported change from baseline: 4-11.6 (range – non-significant); increase of 3.5 from baseline (significant); increase in two dose groups 19 (ABO 2.5 mL) and 25 (ABO 1.5 mL) (significant); age appraisal (2 studies): 0.75 yrs younger and decrease in two dose groups (2.2 yrs in ABO 2.5 mL and 2.0 yrs in ABO 1.5 mL group) (all significant) and age satisfaction (1 study): increases across 3 dose groups: (20U) 19% increase, (60 U) 35% increase, 20U) 34% increase (all non-significant)  Combined treatment: N=6 psychological wellbeing (4 studies): 1 study ONA (Onabotulinum toxin A); multiple sites, 18.2% increase (non-significant); 1 study, ONA and filler, 34.6% increase (significant); 1 study filler and ONA, 19.9% increase (significant); 1 study filler and ONA (high and low sites) 8.2, 5.1% increase (non-significant); social functioning and social confidence (3 studies): 2 studies filler and ONA, 26.9%, 18.2% increase (significant), 1 study filler and ONA (high and low sites) 7.3%, 1.2% increase (non-significant); age appraisal (2 studies): filler and ONA 4.6 yrs younger (non-significant), filler and ONA (high and low sites) 6.3, 3.4 yrs younger (non-significant)  Other treatments: Age-appraisal changes (e.g., PRP, PRF, PRP+laser, microneedling, threading)  Non-significant decreases in age appraisal (decreased perception of individual age) from participating patients  Limitations:  Varying follow-up times; lack of control groups; publication bias; No CA process; No double title screen or DE |
| Imadojemu et al. (2013) | Systematic Review | To examine relevant psychosocial domains of patients after surgical or minimally invasive facial cosmetic  procedures | Total (N=16 studies); N=2 minimally invasive  Jurisdiction: 5 (31%) were from the US &  Canada, others were from 8 different countries | N=53 - 259,  More females than males | Total of 9 procedures included: rhytidectomyBoNT-A, rhinoplasty blepharoplasyotoplasty, ody contouring, filler, laser, cervical liposuction and multiple | Pre-post treatment and placebo  QOL Measures: self-esteem, and body image (Quality of Life Enjoyment and  Satisfaction Questionnaire - QOL-ESQ); enjoyment and satisfaction QoL; self esteem  by HPSSES  Follow up: 3 - 60 months | Laser resurfacing: 1 study reported significant improvement from baseline scores through 6 months post (note, authors presented data in aggregate and did not comment on laser resurfacing in particular)  Botox: Significant improvement in overall life satisfaction and self- esteem through 3 months compared to placebo.  Limitations:  Heterogeneity of studies, lack of minimally invasive studies (only 1 high-quality, procedure specific study investigating minimally invasive procedures),  dearth of rigorous data |
| Ou et al. (2023) | Systematic Review | To identify chin augmentation  injection techniques used across studies and patient satisfaction and complications | Total (N=8)  Jurisdiction:  Not reported | N=917  Age= 18-80 years; Female=795 (86.7%) | Chin augmentation (non-surgical) with hyaluronic acid (HA) filler | Pre-post treatment  QoL Measures:  Most studies used either the  Global Aesthetic Improvement Scale (GAIS) or  FACE-Q questionnaire (others used unnamed point scales of improvement and patient satisfaction)  Follow-up: 2 weeks - 12 months | For GAIS, the proportion of participants with  improved/much improved scores varied between 77.2 and 99%.  For FACE-Q, patient scores from baseline greatly improved in both chin and psychosocial wellbeing  Limitations:  Significant heterogeneity of injection technique, assessment methods, outcomes and complications may not be reliably compared, most studies had short follow-up and only reported events could be taken into the analysis; Reported Level III evidence without reference to specific CA tool |
| Shah & Rieder (2021) | Systematic Review | To systematically  review the evidence concerning observer reported outcomes (OROs) after cosmetic procedures | Total (N=24)  Jurisdiction:  Not reported | N=686 patients  N=8,257 observers  Mean age=  49.1 (11 to 73 years), mostly female | Interventions included both invasive and non-invasive. Treatments A total of 6 non-invasive treatments were included:  botulinum toxin, calcium hydroxyapatite, hyaluronic  acid, deoxycholic acid, fat injection, laser skin resurfacing | Pre-post treatment and control groups  QoL Measures:  Measured OROs on aesthetics and wellness, social capacities, and skills and competencies  Most studies self-developed an ORO tool; multiple studies used FIQ (First Impression  Questionnaire) | Aesthetics & Wellness:  Age, attractiveness, and health consistently demonstrated statistical significance in improved  post-procedure outcomes across several invasive and non-invasive treatments – the majority high quality studies of minimally invasive treatments reported statistical significance for attractiveness  Perceived  sexual dimorphism, health, and overall first impression demonstrated statistical significance; however, limited evidence reduces generalizability  Social Capacities:  Results suggest augmented  perception of social skills, reduced social anxiety, increased likeability, increased friendliness, and increased approachability – no statistical significance within the domain of sociability, perception of  extroversion and kindness  Several non-invasive studies report increased perceived success (at relationships) post-treatment  Significant increases for perceived trustworthiness and confidence; however, limited evidence reduces generalizability  Limitations:  Reported low-moderate levels of evidence without reference to specific CA tool; differing questionnaires used led to difficult assessment across studies; longer follow-up times required; need for untreated control groups |
| Wang & Rieder (2019) | Systematic Review | To summarize the tools and outcomes in evaluating patient QoL after aesthetic Botox administration | Total (N=50 studies) (46 unique study populations)  Jurisdiction:  Not reported | N=11-1021 | Botox for several treatment areas | N=17 Placebo; N=23 None; N=10 Other treatment  QoL Measures:  Likert scales (5 types)  FTS (Facial Lines Treatment), FLO (Facial Lines Outcome), SPA (Service Provision Assessment), FACE-Q  Follow-up: 1 week-26 months | Increases in positive appearance, perception of youthfulness, attractiveness, restedness, affect, mood, and self-confidence  Combination treatments, can increase both QoL and patient satisfaction  Limitations:  More diverse study population; patient satisfaction may not be correlated with QoL |
